# 3-(2-Benzamido­phen­yl)-4-(4-hydroxy­phen­yl)-5-methyl-4*H*-1,2,4-triazol-1-ium chloride

**DOI:** 10.1107/S160053680802148X

**Published:** 2008-07-16

**Authors:** Mohammad Arfan, M. Nawaz Tahir, Rasool Khan, Mohammad S. Iqbal

**Affiliations:** a Institute of Chemical Sciences, University of Peshawar, Peshawar, Pakistan; b Department of Physics, University of Sargodha, Sargodha, Pakistan; c Department of Chemistry, Government College University, Lahore, Pakistan

## Abstract

In the mol­ecule of the title compound, C_22_H_19_N_4_O_2_^+^·Cl^−^, the 1,2,4-triazole ring is oriented at dihedral angles of 75.57 (14), 53.23 (13) and 68.11 (13)° with respect to the benzamide, aniline and phenol atomatic rings, respectively. An intra­molecular C—H⋯O hydrogen bond results in the formation of a non-planar ten-membered ring. In the crystal structure, inter­molecular O—H⋯Cl and N—H⋯Cl hydrogen bonds link the mol­ecules. There is a C—H⋯π contact between the methyl group and the phenyl ring, and a π–π contact between the hydroxy­phenyl and phenyl rings [centroid–centroid distance = 4.687 (2) Å].

## Related literature

For general background, see: Caira *et al.* (2004 ▶); Peeters *et al.* (1996 ▶). For related literature, see: Potts (1961 ▶).
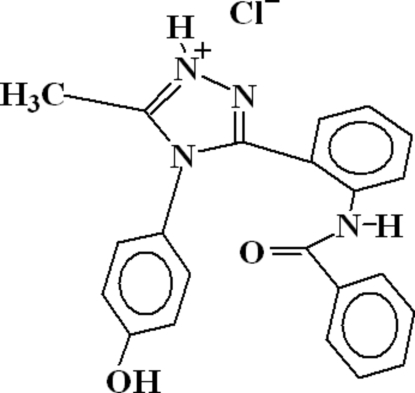


## Experimental

### 

#### Crystal data

C_22_H_19_N_4_O_2_^+^·Cl^−^
*M* *_r_* = 406.86
Orthorhombic, 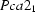

*a* = 17.1336 (15) Å
*b* = 9.8967 (9) Å
*c* = 12.1910 (10) Å
*V* = 2067.2 (3) Å^3^
*Z* = 4
Mo *K*α radiation
μ = 0.21 mm^−1^
*T* = 296 (2) K
0.25 × 0.20 × 0.18 mm

#### Data collection

Bruker Kappa APEXII CCD diffractometer
Absorption correction: multi-scan (*SADABS*; Bruker, 2005 ▶) *T*_min_ = 0.950, *T*_max_ = 0.960
13329 measured reflections
2809 independent reflections
2480 reflections with *I* > 2σ(*I*)
*R*_int_ = 0.022

#### Refinement

*R*[*F* ^2^ > 2σ(*F* ^2^)] = 0.034
*wR*(*F* ^2^) = 0.097
*S* = 1.02
2809 reflections
272 parameters
1 restraint
H atoms treated by a mixture of independent and constrained refinement
Δρ_max_ = 0.20 e Å^−3^
Δρ_min_ = −0.21 e Å^−3^
Absolute structure: Flack (1983 ▶), with 1677 Friedel pairs
Flack parameter: 0.06 (7)

### 

Data collection: *APEX2* (Bruker, 2007 ▶); cell refinement: *APEX2*; data reduction: *SAINT* (Bruker, 2007 ▶); program(s) used to solve structure: *SHELXS97* (Sheldrick, 2008 ▶); program(s) used to refine structure: *SHELXL97* (Sheldrick, 2008 ▶); molecular graphics: *ORTEP-3 for Windows* (Farrugia, 1997 ▶) and *PLATON* (Spek, 2003 ▶); software used to prepare material for publication: *WinGX* (Farrugia, 1999 ▶) and *PLATON*.

## Supplementary Material

Crystal structure: contains datablocks global, I. DOI: 10.1107/S160053680802148X/hk2490sup1.cif

Structure factors: contains datablocks I. DOI: 10.1107/S160053680802148X/hk2490Isup2.hkl

Additional supplementary materials:  crystallographic information; 3D view; checkCIF report

## Figures and Tables

**Table 1:** Hydrogen-bond geometry (Å, °) *Cg*2 is the centroid of the C1–C6 phenyl ring.

*D*—H⋯*A*	*D*—H	H⋯*A*	*D*⋯*A*	*D*—H⋯*A*
N1—H1⋯Cl^i^	0.81 (3)	2.58 (3)	3.372 (2)	166 (3)
O2—H2*A*⋯Cl^ii^	0.76 (4)	2.34 (4)	3.090 (2)	171 (5)
N3—H3*A*⋯Cl^iii^	0.97 (3)	2.14 (3)	3.068 (2)	158 (3)
C18—H18⋯O1	0.93	2.35	3.165 (3)	146
C16—H16*A*⋯*Cg*2^iv^	0.96	2.84	3.367 (3)	115

Symmetry codes: (i) 
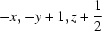
; (ii) 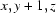; (iii) 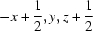; (iv) 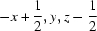.

**Fig. 1.**
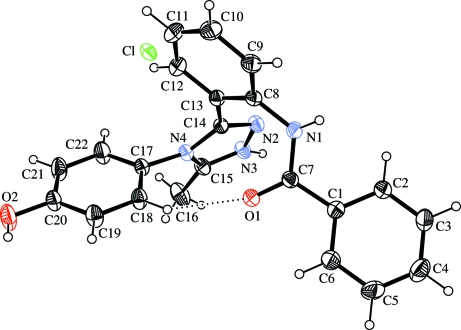
The molecular structure of the title molecule, with the atom-numbering scheme. Displacement ellipsoids are drawn at the 30% probability level. Hydrogen bond is shown as dotted line.

**Fig. 2.**
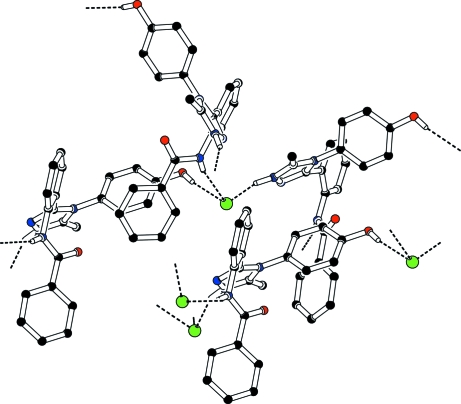
A partial packing diagram of the title compound. Hydrogen bonds are shown as dashed lines.

## supplementary crystallographic information

### Comment

1,2,4-Triazole is a basic aromatic heterocycle. Its derivatives, such as fluconazole (Caira *et al.*,  2004) and itraconazole (Peeters *et al.*,  1996), find use as antifungals and can be prepared using various routes including Einhorn-Brunner and Pellizzari reactions (Potts, 1961).

In the molecule of the title compound (Fig. 1) the bond lengths and angles are generally within normal ranges. Rings A (C1-C6), B (C8-C13), C (N2-N4/C14/C15) and D (C17-C22) are, of course, planar, and the dihedral angles between them are A/B = 66.39 (14)°, A/C = 75.57 (14)°, A/D = 39.96 (13)°, B/C = 53.23 (13)°, B/D = 78.57 (14)° and C/D = 68.11 (13)°. The intramolecular C-H···O hydrogen bond (Table 1) results in the formation of a non-planar ten-membered ring E (O1/N1/N4/C7/C8/C13/C14/C17/C18/H18).

In the crystal structure, intermolecular O-H···Cl and N-H···Cl hydrogen bonds (Table 1) link the molecules (Fig. 2), in which they may be effective in the stabilization of the structure. The C—H···π contact (Table 1) between the methyl group and the phenyl ring and π—π contact between the two phenyl rings Cg2···Cg4^i^ [symmetry code: (i) -x, 2 - y, 1/2 + z, where Cg2 and Cg4 are centroids of the rings (C1-C6) and (C17-C22), respectively] further stabilize the structure, with centroid-centroid distance of 4.687 (2) Å.

### Experimental

For the preparation of the title compound, N-[2-(hydrazinylcarbonyl)phenyl]- benzamide (0.25 g, 0.98 mmol) and triethyl orthoacetate (1 ml) were refluxed for 2 h. The reaction mixture was cooled to room temperature and 4-aminophenol (0.11 g, 0.98 mmol) was added. Acetic acid (1 ml) was added to the mixture and the contents were refluxed for 1 h. Then, the reaction mixture was added to HCl (8 M) solution. It was crystallized by adding dioxane portion-wise. The crystals were separated by filtration and washed with dioxane (yield; 77%, m.p. 498 K).

### Refinement

H atoms, H2A (for OH), H1 and H3A (for NH), were located in difference syntheses and refined [O-H = 0.76 (4) Å, U_iso_(H) = 0.0839 Å^2^ and N-H = 0.81 (3) and 0.97 (3) Å, U_iso_(H) = 0.0628 and 0.0492 Å^2^]. The remaining H atoms were positioned geometrically, with C-H = 0.93 and 0.96 Å for aromatic and methyl H, respectively, and constrained to ride on their parent atoms with U_iso_(H) = xU_eq_(C), where x = 1.5 for methyl H and x = 1.2 for aromatic H atoms.

### Crystal data

**Table:**

C_22_H_19_N_4_O_2_^+^·Cl^–^	*F*_000_ = 848
*M**_r_* = 406.86	*D*_x_ = 1.307 Mg m^−^^3^
Orthorhombic, *P**c**a*2_1_	Mo *K*α radiation λ = 0.71073 Å
Hall symbol: P 2c -2ac	Cell parameters from 2009 reflections
*a* = 17.1336 (15) Å	θ = 2.1–28.8º
*b* = 9.8967 (9) Å	µ = 0.21 mm^−^^1^
*c* = 12.1910 (10) Å	*T* = 296 (2) K
*V* = 2067.2 (3) Å^3^	Prismatic, black
*Z* = 4	0.25 × 0.20 × 0.18 mm

### Data collection

**Table:**

Bruker Kappa APEXII CCD diffractometer	2809 independent reflections
Radiation source: fine-focus sealed tube	2480 reflections with *I* > 2σ(*I*)
Monochromator: graphite	*R*_int_ = 0.022
Detector resolution: 7.40 pixels mm^-1^	θ_max_ = 28.8º
*T* = 296(2) K	θ_min_ = 2.1º
ω scans	*h* = −23→18
Absorption correction: multi-scan(SADABS; Bruker, 2005)	*k* = −13→10
*T*_min_ = 0.950, *T*_max_ = 0.960	*l* = −16→12
13329 measured reflections	

### Refinement

**Table:**

Refinement on *F*^2^	Hydrogen site location: inferred from neighbouring sites
Least-squares matrix: full	H atoms treated by a mixture of independent and constrained refinement
*R*[*F*^2^ > 2σ(*F*^2^)] = 0.034	*w* = 1/[σ^2^(*F*_o_^2^) + (0.0606*P*)^2^ + 0.2556*P*] where *P* = (*F*_o_^2^ + 2*F*_c_^2^)/3
*wR*(*F*^2^) = 0.097	(Δ/σ)_max_ = 0.001
*S* = 1.02	Δρ_max_ = 0.20 e Å^−^^3^
2809 reflections	Δρ_min_ = −0.21 e Å^−^^3^
272 parameters	Extinction correction: none
1 restraint	Absolute structure: Flack (1983) with no Friedel pairs
Primary atom site location: structure-invariant direct methods	Flack parameter: 0.06 (7)
Secondary atom site location: difference Fourier map	

### Special details

**Table:**

Geometry. Bond distances, angles etc have been calculated using the rounded fractional coordinates. All su's are estimated from the variances of the (full) variance-covariance matrix. The cell e.s.d.'s are taken into account in the estimation of distances, angles and torsion angles
Refinement. Refinement of F^2^ against ALL reflections. The weighted R-factor wR and goodness of fit S are based on F^2^, conventional R-factors R are based on F, with F set to zero for negative F^2^. The threshold expression of F^2^ > 2sigma(F^2^) is used only for calculating R-factors(gt) etc. and is not relevant to the choice of reflections for refinement. R-factors based on F^2^ are statistically about twice as large as those based on F, and R- factors based on ALL data will be even larger.

### Fractional atomic coordinates and isotropic or equivalent isotropic displacement parameters (Å^2^)

**Table:**

	*x*	*y*	*z*	*U*_iso_*/*U*_eq_	
Cl	0.18901 (3)	0.41678 (5)	0.23611 (6)	0.0441 (2)	
O1	0.08185 (11)	0.8416 (2)	0.61956 (15)	0.0523 (6)	
O2	0.23156 (16)	1.1272 (2)	0.1626 (2)	0.0698 (9)	
N1	−0.02194 (12)	0.7017 (2)	0.62595 (16)	0.0396 (6)	
N2	0.12701 (11)	0.54036 (19)	0.55257 (17)	0.0394 (5)	
N3	0.20680 (12)	0.5419 (2)	0.55843 (18)	0.0410 (6)	
N4	0.17842 (10)	0.69261 (19)	0.44104 (16)	0.0345 (5)	
C1	0.02424 (13)	0.8077 (2)	0.79409 (19)	0.0364 (6)	
C2	−0.01415 (15)	0.7183 (3)	0.8629 (2)	0.0451 (8)	
C3	−0.02023 (18)	0.7468 (4)	0.9741 (2)	0.0583 (10)	
C4	0.0117 (2)	0.8628 (3)	1.0166 (3)	0.0640 (10)	
C5	0.0506 (2)	0.9501 (3)	0.9485 (3)	0.0648 (10)	
C6	0.05751 (18)	0.9231 (3)	0.8372 (2)	0.0513 (8)	
C7	0.03122 (13)	0.7860 (2)	0.6732 (2)	0.0364 (6)	
C8	−0.03057 (13)	0.6865 (2)	0.51128 (19)	0.0357 (6)	
C9	−0.10597 (14)	0.6966 (3)	0.4680 (2)	0.0477 (8)	
C10	−0.11851 (17)	0.6838 (3)	0.3569 (3)	0.0580 (9)	
C11	−0.05706 (19)	0.6642 (3)	0.2866 (2)	0.0590 (10)	
C12	0.01801 (16)	0.6538 (3)	0.3276 (2)	0.0476 (8)	
C13	0.03172 (13)	0.6628 (2)	0.44022 (18)	0.0352 (6)	
C14	0.11081 (12)	0.6336 (2)	0.48077 (18)	0.0340 (6)	
C15	0.23791 (13)	0.6310 (2)	0.4926 (2)	0.0391 (6)	
C16	0.32228 (14)	0.6578 (3)	0.4792 (3)	0.0637 (10)	
C17	0.18724 (12)	0.8055 (2)	0.36715 (19)	0.0348 (6)	
C18	0.16505 (16)	0.9329 (2)	0.3999 (2)	0.0441 (7)	
C19	0.17852 (16)	1.0418 (3)	0.3320 (2)	0.0497 (8)	
C20	0.21503 (14)	1.0236 (2)	0.2313 (2)	0.0456 (7)	
C21	0.2375 (2)	0.8955 (3)	0.2002 (2)	0.0612 (9)	
C22	0.22377 (19)	0.7867 (3)	0.2674 (2)	0.0575 (9)	
H1	−0.061 (2)	0.684 (3)	0.661 (3)	0.0628*	
H2	−0.03578	0.63946	0.83474	0.0541*	
H2A	0.220 (2)	1.195 (4)	0.187 (4)	0.0839*	
H3	−0.04612	0.68676	1.02018	0.0700*	
H3A	0.2334 (17)	0.480 (3)	0.608 (3)	0.0492*	
H4	0.00691	0.88200	1.09092	0.0768*	
H5	0.07271	1.02826	0.97730	0.0778*	
H6	0.08444	0.98261	0.79188	0.0616*	
H9	−0.14797	0.71205	0.51465	0.0573*	
H10	−0.16905	0.68850	0.32930	0.0696*	
H11	−0.06583	0.65794	0.21149	0.0710*	
H12	0.05962	0.64081	0.27972	0.0571*	
H16A	0.33112	0.75352	0.47749	0.0956*	
H16B	0.35037	0.61882	0.53950	0.0956*	
H16C	0.34011	0.61830	0.41172	0.0956*	
H18	0.14104	0.94552	0.46751	0.0529*	
H19	0.16312	1.12783	0.35368	0.0596*	
H21	0.26212	0.88257	0.13302	0.0734*	
H22	0.23907	0.70060	0.24582	0.0689*	

### Atomic displacement parameters (Å^2^)

**Table:**

	*U*^11^	*U*^22^	*U*^33^	*U*^12^	*U*^13^	*U*^23^
Cl	0.0360 (2)	0.0416 (3)	0.0547 (3)	−0.0066 (2)	0.0002 (3)	0.0095 (3)
O1	0.0540 (10)	0.0641 (11)	0.0389 (9)	−0.0263 (9)	0.0049 (8)	−0.0019 (9)
O2	0.104 (2)	0.0464 (10)	0.0590 (13)	0.0042 (12)	0.0244 (13)	0.0210 (11)
N1	0.0295 (9)	0.0549 (12)	0.0343 (10)	−0.0086 (8)	0.0027 (7)	−0.0004 (9)
N2	0.0373 (9)	0.0381 (9)	0.0428 (10)	−0.0018 (8)	−0.0011 (8)	0.0070 (9)
N3	0.0360 (9)	0.0375 (9)	0.0494 (11)	0.0008 (8)	−0.0005 (9)	0.0105 (9)
N4	0.0345 (9)	0.0309 (9)	0.0382 (10)	0.0006 (7)	0.0047 (7)	0.0034 (7)
C1	0.0332 (10)	0.0399 (11)	0.0362 (11)	0.0028 (8)	−0.0017 (9)	0.0000 (9)
C2	0.0452 (13)	0.0516 (14)	0.0386 (12)	−0.0102 (10)	−0.0013 (10)	0.0019 (11)
C3	0.0594 (16)	0.075 (2)	0.0406 (13)	−0.0120 (14)	0.0071 (12)	0.0054 (14)
C4	0.083 (2)	0.0655 (18)	0.0435 (14)	0.0084 (16)	0.0101 (15)	−0.0096 (14)
C5	0.094 (2)	0.0415 (13)	0.0588 (18)	−0.0032 (14)	0.0028 (17)	−0.0158 (13)
C6	0.0678 (17)	0.0380 (12)	0.0481 (14)	−0.0035 (11)	0.0037 (13)	0.0006 (10)
C7	0.0334 (11)	0.0384 (11)	0.0373 (11)	−0.0034 (8)	−0.0004 (9)	0.0022 (9)
C8	0.0325 (10)	0.0392 (11)	0.0353 (10)	−0.0063 (8)	−0.0030 (8)	0.0015 (9)
C9	0.0326 (10)	0.0598 (15)	0.0508 (14)	−0.0082 (10)	−0.0051 (10)	0.0026 (12)
C10	0.0462 (14)	0.0737 (18)	0.0540 (16)	−0.0104 (13)	−0.0210 (13)	0.0084 (14)
C11	0.0653 (18)	0.075 (2)	0.0367 (13)	−0.0117 (15)	−0.0159 (12)	0.0048 (13)
C12	0.0534 (14)	0.0549 (14)	0.0346 (12)	−0.0025 (11)	−0.0022 (10)	0.0006 (11)
C13	0.0366 (10)	0.0353 (10)	0.0337 (10)	−0.0039 (8)	−0.0027 (8)	0.0040 (9)
C14	0.0359 (10)	0.0325 (10)	0.0336 (10)	−0.0028 (8)	0.0028 (8)	0.0009 (8)
C15	0.0369 (12)	0.0323 (9)	0.0482 (12)	0.0022 (8)	0.0032 (10)	0.0050 (10)
C16	0.0338 (13)	0.0642 (18)	0.093 (2)	0.0031 (11)	0.0058 (14)	0.0277 (18)
C17	0.0365 (10)	0.0309 (10)	0.0369 (10)	0.0006 (8)	0.0046 (9)	0.0054 (9)
C18	0.0518 (13)	0.0379 (12)	0.0425 (12)	0.0099 (10)	0.0153 (11)	0.0057 (10)
C19	0.0625 (16)	0.0339 (11)	0.0527 (14)	0.0139 (10)	0.0168 (13)	0.0069 (11)
C20	0.0533 (12)	0.0394 (11)	0.0440 (12)	0.0020 (9)	0.0076 (12)	0.0113 (11)
C21	0.091 (2)	0.0481 (14)	0.0446 (14)	0.0031 (14)	0.0306 (15)	0.0036 (11)
C22	0.086 (2)	0.0351 (12)	0.0515 (16)	0.0072 (12)	0.0283 (14)	0.0009 (10)

### Geometric parameters (Å, °)

**Table:**

O1—C7	1.218 (3)	C8—C9	1.399 (3)
O2—C20	1.354 (3)	C8—C13	1.395 (3)
O2—H2A	0.76 (4)	C9—C10	1.377 (4)
N1—C7	1.363 (3)	C9—H9	0.9300
N1—C8	1.414 (3)	C10—C11	1.371 (4)
N1—H1	0.81 (3)	C10—H10	0.9300
N2—N3	1.369 (3)	C11—C12	1.384 (4)
N2—C14	1.302 (3)	C11—H11	0.9300
N3—C15	1.306 (3)	C12—C13	1.396 (3)
N3—H3A	0.97 (3)	C12—H12	0.9300
N4—C14	1.385 (3)	C13—C14	1.471 (3)
N4—C15	1.344 (3)	C15—C16	1.479 (3)
N4—C17	1.443 (3)	C16—H16A	0.9600
C1—C2	1.385 (3)	C16—H16B	0.9600
C1—C6	1.380 (4)	C16—H16C	0.9600
C1—C7	1.494 (3)	C17—C18	1.376 (3)
C2—C3	1.389 (4)	C17—C22	1.380 (3)
C2—H2	0.9300	C18—C19	1.378 (4)
C3—C4	1.373 (5)	C18—H18	0.9300
C3—H3	0.9300	C19—C20	1.390 (3)
C4—C5	1.371 (5)	C19—H19	0.9300
C4—H4	0.9300	C20—C21	1.378 (4)
C5—C6	1.388 (4)	C21—C22	1.373 (4)
C5—H5	0.9300	C21—H21	0.9300
C6—H6	0.9300	C22—H22	0.9300
			
Cl···O2^i^	3.090 (2)	C21···O1^iii^	3.291 (4)
Cl···N1^ii^	3.372 (2)	C22···C16	3.338 (4)
Cl···C2^ii^	3.627 (3)	C2···H19^ix^	2.9700
Cl···N3^iii^	3.068 (2)	C2···H1	2.61 (4)
Cl···H2^ii^	2.9400	C4···H16A^v^	2.9400
Cl···H9^ii^	3.0700	C5···H16A^v^	2.8300
Cl···H1^ii^	2.58 (3)	C6···H16A^v^	3.0600
Cl···H22	2.9400	C8···H5^vii^	2.9400
Cl···H10^iv^	2.8800	C9···H5^vii^	2.7800
Cl···H2A^i^	2.34 (4)	C12···H2^ii^	2.9200
Cl···H3A^iii^	2.14 (3)	C15···H21^v^	3.0200
O1···N4	3.106 (3)	C15···H22	3.0900
O1···N2	3.187 (3)	C17···H16A	2.8600
O1···C18	3.165 (3)	C17···H12	2.9300
O1···C13	2.941 (3)	H1···H9	2.3400
O1···C14	2.710 (3)	H1···C2	2.61 (4)
O1···C21^v^	3.291 (4)	H1···Cl^viii^	2.58 (3)
O2···Cl^vi^	3.090 (2)	H1···H2	2.2100
O1···H6	2.5200	H2···N1	2.6300
O1···H18	2.3500	H2···H1	2.2100
O1···H21^v^	2.7100	H2···C12^viii^	2.9200
O2···H9^vii^	2.8000	H2···Cl^viii^	2.9400
N1···Cl^viii^	3.372 (2)	H2A···H19	2.3500
N1···N2	3.141 (3)	H2A···Cl^vi^	2.34 (4)
N2···C7	3.281 (3)	H3···H11^x^	2.3700
N2···N4	2.212 (3)	H3···N2^viii^	2.6700
N2···O1	3.187 (3)	H3A···H16B	2.5700
N2···N1	3.141 (3)	H3A···Cl^v^	2.14 (3)
N3···Cl^v^	3.068 (2)	H5···C9^ix^	2.7800
N3···N4	2.124 (3)	H5···C8^ix^	2.9400
N4···N3	2.124 (3)	H6···O1	2.5200
N4···O1	3.106 (3)	H9···Cl^viii^	3.0700
N1···H2	2.6300	H9···O2^ix^	2.8000
N2···H3^ii^	2.6700	H9···H1	2.3400
N3···H22^v^	2.9200	H10···Cl^xi^	2.8800
N4···H12	2.8800	H11···H3^xii^	2.3700
C2···Cl^viii^	3.627 (3)	H12···N4	2.8800
C3···C16^v^	3.505 (4)	H12···C17	2.9300
C4···C16^v^	3.524 (4)	H16A···C4^iii^	2.9400
C7···N2	3.281 (3)	H16A···C17	2.8600
C7···C14	3.105 (3)	H16A···C5^iii^	2.8300
C12···C17	3.301 (3)	H16A···C6^iii^	3.0600
C13···O1	2.941 (3)	H16B···H3A	2.5700
C13···C18	3.550 (3)	H18···O1	2.3500
C14···O1	2.710 (3)	H19···C2^vii^	2.9700
C14···C7	3.105 (3)	H19···H2A	2.3500
C16···C22	3.338 (4)	H21···C15^iii^	3.0200
C16···C4^iii^	3.524 (4)	H21···O1^iii^	2.7100
C16···C3^iii^	3.505 (4)	H22···C15	3.0900
C17···C12	3.301 (3)	H22···N3^iii^	2.9200
C18···C13	3.550 (3)	H22···Cl	2.9400
C18···O1	3.165 (3)		
			
C20—O2—H2A	112 (3)	N4—C17—C18	120.0 (2)
C7—N1—C8	123.6 (2)	C17—C18—C19	119.7 (2)
N3—N2—C14	103.89 (18)	C18—C19—C20	120.3 (2)
N2—N3—C15	112.5 (2)	O2—C20—C19	122.9 (2)
C14—N4—C15	106.23 (18)	C19—C20—C21	119.2 (2)
C14—N4—C17	129.23 (18)	O2—C20—C21	117.9 (2)
C15—N4—C17	124.32 (18)	C20—C21—C22	120.6 (2)
C8—N1—H1	114 (3)	C17—C22—C21	119.8 (3)
C7—N1—H1	117 (2)	C1—C2—H2	120.00
N2—N3—H3A	119.5 (18)	C3—C2—H2	120.00
C15—N3—H3A	128.0 (18)	C2—C3—H3	120.00
C2—C1—C6	119.6 (2)	C4—C3—H3	120.00
C2—C1—C7	122.9 (2)	C3—C4—H4	120.00
C6—C1—C7	117.5 (2)	C5—C4—H4	120.00
C1—C2—C3	119.8 (3)	C4—C5—H5	120.00
C2—C3—C4	120.6 (3)	C6—C5—H5	120.00
C3—C4—C5	119.5 (3)	C1—C6—H6	120.00
C4—C5—C6	120.8 (3)	C5—C6—H6	120.00
C1—C6—C5	119.8 (3)	C8—C9—H9	120.00
N1—C7—C1	116.83 (19)	C10—C9—H9	120.00
O1—C7—N1	121.7 (2)	C9—C10—H10	120.00
O1—C7—C1	121.5 (2)	C11—C10—H10	120.00
C9—C8—C13	119.0 (2)	C10—C11—H11	120.00
N1—C8—C13	123.5 (2)	C12—C11—H11	120.00
N1—C8—C9	117.5 (2)	C11—C12—H12	120.00
C8—C9—C10	120.6 (2)	C13—C12—H12	120.00
C9—C10—C11	120.5 (3)	C15—C16—H16A	109.00
C10—C11—C12	119.9 (2)	C15—C16—H16B	109.00
C11—C12—C13	120.5 (2)	C15—C16—H16C	109.00
C12—C13—C14	118.2 (2)	H16A—C16—H16B	110.00
C8—C13—C12	119.5 (2)	H16A—C16—H16C	109.00
C8—C13—C14	122.0 (2)	H16B—C16—H16C	109.00
N2—C14—N4	110.84 (18)	C17—C18—H18	120.00
N2—C14—C13	124.17 (19)	C19—C18—H18	120.00
N4—C14—C13	124.76 (19)	C18—C19—H19	120.00
N3—C15—N4	106.5 (2)	C20—C19—H19	120.00
N4—C15—C16	127.5 (2)	C20—C21—H21	120.00
N3—C15—C16	126.0 (2)	C22—C21—H21	120.00
N4—C17—C22	119.5 (2)	C17—C22—H22	120.00
C18—C17—C22	120.3 (2)	C21—C22—H22	120.00
			
C8—N1—C7—O1	−9.5 (3)	C1—C2—C3—C4	−0.2 (4)
C8—N1—C7—C1	170.21 (19)	C2—C3—C4—C5	−0.7 (5)
C7—N1—C8—C9	−130.2 (2)	C3—C4—C5—C6	0.5 (5)
C7—N1—C8—C13	49.5 (3)	C4—C5—C6—C1	0.6 (5)
C14—N2—N3—C15	−0.6 (3)	N1—C8—C9—C10	179.5 (2)
N3—N2—C14—N4	0.5 (2)	C13—C8—C9—C10	−0.2 (4)
N3—N2—C14—C13	175.2 (2)	N1—C8—C13—C12	−177.8 (2)
N2—N3—C15—N4	0.4 (3)	N1—C8—C13—C14	8.3 (3)
N2—N3—C15—C16	−179.9 (2)	C9—C8—C13—C12	1.9 (3)
C15—N4—C14—N2	−0.2 (2)	C9—C8—C13—C14	−172.0 (2)
C15—N4—C14—C13	−174.9 (2)	C8—C9—C10—C11	−1.6 (4)
C17—N4—C14—N2	−175.0 (2)	C9—C10—C11—C12	1.6 (5)
C17—N4—C14—C13	10.4 (3)	C10—C11—C12—C13	0.2 (4)
C14—N4—C15—N3	−0.1 (2)	C11—C12—C13—C8	−1.9 (4)
C14—N4—C15—C16	−179.8 (2)	C11—C12—C13—C14	172.2 (2)
C17—N4—C15—N3	175.0 (2)	C8—C13—C14—N2	52.0 (3)
C17—N4—C15—C16	−4.7 (4)	C8—C13—C14—N4	−134.1 (2)
C14—N4—C17—C18	67.0 (3)	C12—C13—C14—N2	−122.0 (3)
C14—N4—C17—C22	−118.2 (3)	C12—C13—C14—N4	52.0 (3)
C15—N4—C17—C18	−106.9 (3)	N4—C17—C18—C19	175.6 (2)
C15—N4—C17—C22	67.8 (3)	C22—C17—C18—C19	0.9 (4)
C6—C1—C2—C3	1.3 (4)	N4—C17—C22—C21	−175.2 (3)
C7—C1—C2—C3	−178.2 (2)	C18—C17—C22—C21	−0.5 (4)
C2—C1—C6—C5	−1.5 (4)	C17—C18—C19—C20	−0.6 (4)
C7—C1—C6—C5	178.0 (3)	C18—C19—C20—O2	−178.2 (3)
C2—C1—C7—O1	−159.0 (2)	C18—C19—C20—C21	0.1 (4)
C2—C1—C7—N1	21.3 (3)	O2—C20—C21—C22	178.7 (3)
C6—C1—C7—O1	21.5 (3)	C19—C20—C21—C22	0.3 (4)
C6—C1—C7—N1	−158.2 (2)	C20—C21—C22—C17	−0.1 (5)

Symmetry codes: (i) *x*, *y*−1, *z*; (ii) −*x*, −*y*+1, *z*−1/2; (iii) −*x*+1/2, *y*, *z*−1/2; (iv) *x*+1/2, −*y*+1, *z*; (v) −*x*+1/2, *y*, *z*+1/2; (vi) *x*, *y*+1, *z*; (vii) −*x*, −*y*+2, *z*−1/2; (viii) −*x*, −*y*+1, *z*+1/2; (ix) −*x*, −*y*+2, *z*+1/2; (x) *x*, *y*, *z*+1; (xi) *x*−1/2, −*y*+1, *z*; (xii) *x*, *y*, *z*−1.

### Hydrogen-bond geometry (Å, °)

**Table:**

*D*—H···*A*	*D*—H	H···*A*	*D*···*A*	*D*—H···*A*
N1—H1···Cl^viii^	0.81 (3)	2.58 (3)	3.372 (2)	166 (3)
O2—H2A···Cl^vi^	0.76 (4)	2.34 (4)	3.090 (2)	171 (5)
N3—H3A···Cl^v^	0.97 (3)	2.14 (3)	3.068 (2)	158 (3)
C18—H18···O1	0.9300	2.3500	3.165 (3)	146.00
C16—H16A···Cg2^iii^	0.9600	2.8400	3.367 (3)	115.00

Symmetry codes: (viii) −*x*, −*y*+1, *z*+1/2; (vi) *x*, *y*+1, *z*; (v) −*x*+1/2, *y*, *z*+1/2; (iii) −*x*+1/2, *y*, *z*−1/2.
